# Supplementary material for: From sequence to enzyme mechanism using multi-label machine learning
Source: BMC Bioinformatics. 2014 May 19;15:150. doi: 10.1186/1471-2105-15-150 (PMC4229970; doi:10.1186/1471-2105-15-150)
Supplement: Additional file 2 — Java code of ml2db. Additional file ml2db_code.tar.gz contains the Java source code to run the multi-label machine learning experiments and save the results to database. The code’s Javadoc is included. [file 1471-2105-15-150-S2.zip › additional file 2/ml2db/utils/doc/uk/ac/ed/inf/utils/database/class-use/TableManager.html]

Uses of Class uk.ac.ed.inf.utils.database.TableManager


---


|  |  |  |
| --- | --- | --- |
| **Overview** \| **Package** \| **Class** \| **Use** \| **Tree** \| **Deprecated** \| **Index** \| **Help** | |  |
| PREV   NEXT | **FRAMES**    **NO FRAMES**     **All Classes** |  |


---


## **Uses of Class uk.ac.ed.inf.utils.database.TableManager**

| Packages that use TableManager | |
| --- | --- |
| **test.database** |  |
| **uk.ac.ed.inf.utils.database** |  |

Uses of TableManager in test.database

| Methods in test.database that return TableManager | |
| --- | --- |
| `static TableManager` | `TableManagerTest.getTableManagerWithDbManagerString()` |
| `static TableManager` | `TableManagerTest.getTableManagerWithDbManagerTable()` |

Uses of TableManager in uk.ac.ed.inf.utils.database

| Methods in uk.ac.ed.inf.utils.database that return TableManager | |
| --- | --- |
| `TableManager` | `TableRow.getTableManager()` |
| `TableManager` | `TableRowSaver.getTableManager()` |
| `TableManager` | `TableManaged.getTableManager()` |
| `TableManager` | `Table.getTableManager(DbManager manager)` |
| `TableManager` | `DbManager.getTableManager(java.lang.String tableName)` |
| `TableManager` | `DbManager.getTableManager(Table table)` |

| Methods in uk.ac.ed.inf.utils.database with parameters of type TableManager | |
| --- | --- |
| `TableRow` | `Table.createRow(java.lang.Object[] values, TableManager manager)` |

Constructors in uk.ac.ed.inf.utils.database with parameters of type TableManager

`TableCreator(TableManager tableManager,
Table table)`

`TableManaged(DbManager dbManager,
TableManager tableManager)`

`TableReader(TableManager tableManager)`

`TableRow(TableManager manager)`

`TableRow(TableManager manager,
java.util.TreeMap<java.lang.String,java.lang.String> columnToValueMap)`
  
          the auto-generated key of this row in its table (if available)

`TableRow(TableManager manager,
java.util.Vector<java.lang.String> columns,
java.util.Vector<java.lang.String> values)`

`TableWriter(TableManager tableManager)`
  
          Constructor 2: takes the table name and a list of fields

---


|  |  |  |
| --- | --- | --- |
| **Overview** \| **Package** \| **Class** \| **Use** \| **Tree** \| **Deprecated** \| **Index** \| **Help** | |  |
| PREV   NEXT | **FRAMES**    **NO FRAMES**     **All Classes** |  |


---
